# Effects of anastomotic technique on early postoperative outcome in open right‐sided hemicolectomy

**DOI:** 10.1002/bjs5.101

**Published:** 2018-09-27

**Authors:** C. Jurowich, S. Lichthardt, N. Matthes, C. Kastner, I. Haubitz, A. Prock, J. Filser, C.‐T. Germer, A. Wiegering

**Affiliations:** ^1^ Department of General, Visceral and Thoracic Surgery Kreiskliniken Altötting/Burghausen Altötting Germany; ^2^ Department of General, Visceral, Vascular and Paediatric Surgery, University Hospital University of Würzburg Würzburg Germany; ^3^ Comprehensive Cancer Centre Mainfranken University of Würzburg Medical Centre Würzburg Germany; ^4^ Department of Biochemistry and Molecular Biology University of Würzburg Würzburg Germany

## Abstract

**Background:**

Despite recent improvements in colonic cancer surgery, the rate of anastomotic leakage after right hemicolectomy is still around 6–7 per cent. This study examined whether anastomotic technique (handsewn or stapled) after open right hemicolectomy for right‐sided colonic cancer influences postoperative complications.

**Methods:**

Patient data from the German Society for General and Visceral Surgery (StuDoQ) registry from 2010 to 2017 were analysed. Univariable and multivariable analyses were performed. The primary endpoint was anastomotic leakage; secondary endpoints were postoperative ileus, complications and length of postoperative hospital stay (LOS).

**Results:**

A total of 4062 patients who had undergone open right hemicolectomy for colonic cancer were analysed. All patients had an ileocolic anastomosis, 2742 handsewn and 1320 stapled. Baseline characteristics were similar. No significant differences were identified in anastomotic leakage, postoperative ileus, reoperation rate, surgical‐site infection, LOS or death. The stapled group had a significantly shorter duration of surgery and fewer Clavien–Dindo grade I–II complications. In multivariable logistic regression analysis, ASA grade and BMI were found to be significantly associated with postoperative complications such as anastomotic leakage, postoperative ileus and reoperation rate.

**Conclusion:**

Handsewn and stapled ileocolic anastomoses for open right‐sided colonic cancer resections are equally safe. Stapler use was associated with reduced duration of surgery and significantly fewer minor complications.

## Introduction

Colorectal cancer affects more than one million patients per year worldwide, accounting for more than 500 000 deaths annually^1^. Over the past two decades improvements in adjuvant chemotherapy and surgical quality have led to better long‐term survival^2^. Complication rates remain high, however, with 30‐day mortality rates following colonic cancer surgery of up to 10 per cent^3^. In particular, the rate of anastomotic leakage after right hemicolectomy is surprisingly high (6·4–7·5 per cent)^4^ ^5^ compared with that for left hemicolectomy (anastomotic leak rate 1·9–6·5 per cent)^5^ ^6^.

Anastomosis techniques after right hemicolectomy vary widely in clinical practice. Ileocolic anastomoses can be end‐to‐end, end‐to‐side, side‐to‐end or side‐to‐side^7^. They can be handsewn in one or more layers, using interrupted or continuous sutures in a variety of sizes, needle configurations and materials, or stapled using linear or circular proprietary devices. In 2011, a Cochrane systematic review^8^ including 1125 patients with ileocolic anastomosis found a significant advantage for stapled anastomosis with respect to anastomotic leak rate, although studies published subsequently have conversely identified stapled anastomosis as an independent risk factor for anastomotic leakage^9^.

The technical requirements for surgical resection of right‐sided cancers have changed greatly in recent years with the introduction of complete mesocolic excision (CME)^10^, but the optimal anastomotic technique remains an unresolved issue.

Data for colonic cancer were retrieved from the Study, Documentation and Quality Centre (StuDoQ|ColonCancer) registry of the German Society for General and Visceral Surgery (DGAV) to investigate whether the anastomosis techniques influence early postoperative complications.

## Methods

Informed consent and data safety procedures were approved by the Society for Technology, Methods, and Infrastructure for Networked Medical Research (www.tmf‐ev.de), and publication guidelines were established by the DGAV (www.dgav.de/studoq/datenschutzkonzept‐und‐publikationsrichtlinien.html).

The StuDoQ|ColonCancer registry is a voluntary prospectively created database for colonic cancer surgery established by the DGAV in January 2010 (www.dgav.de/studoq; www.en.studoq.de), designed to facilitate assessment of quality and risk factors in colonic cancer surgery in Germany. Data from participating centres are entered in pseudonymized form using a browser‐based tool and subjected to automatic plausibility and cross‐checking controls. Hospitals included in the study data are listed in *Table S1* (supporting information).

For this study, all patients with right‐sided or extended right hemicolectomy were identified from the registry and relevant demographic data, co‐morbidities, and information on operations, histology and perioperative course were extracted in anonymized form for analysis. Patients undergoing emergency surgery, non‐right‐sided resection, laparoscopic right‐sided resection, endoluminal resection, simultaneous liver metastasis resection or creation of any kind of ostomy were excluded. CME should have been performed according to the description by Hohenberger and colleagues^11^. Extended hemicolectomy was defined as any right‐sided colonic resection, including ligation of the middle colic artery and vein.

Anastomotic leakage requiring intervention^12^ ^13^, surgical‐site infection (SSI) necessitating reopening of the wound^14^, Clavien–Dindo complication grade^15^, burst abdomen, reoperation and in‐hospital mortality were evaluated, along with any need for unplanned postoperative ventilation for more than 48 h, pneumonia, length of postoperative hospital stay (LOS) and readmission. Overall postoperative morbidity was summarized according to the Clavien–Dindo classification: grade 0, none; grade I–II, minor; grade IIIa–IV, major; grade V, death.

Patients were grouped according to the type of anastomosis (handsewn or stapled using any type of stapler device). The registry did not contain specific details of anatomical configuration (such as end‐to‐end or side‐to side), suture materials or technique, or the stapling device used.

The primary endpoint was anastomotic leakage. Secondary endpoints were Clavien–Dindo graded postoperative complications, postoperative ileus, reoperation rate, LOS, duration of surgery, 30‐day mortality and MTL30^16^. MTL30 is a new, validated, endpoint parameter specific for the German health sector; it combines mortality, transfer to a higher‐level hospital owing to complications, and length of stay beyond 30 days after surgery.

### Statistical analysis

Statistical analyses are two‐sided, with a significance level of 0·050. Continuous variables are expressed as mean(s.d.) values, and categorical parameters as absolute frequency and percentage. Univariable analysis was performed using χ^2^ and Mann–Whitney *U* tests, as appropriate. Multivariable analysis was by Cox regression. All variables with *P* < 0·100 in univariable analysis were included in the multivariable analysis.

## Results

**Figure 1:**
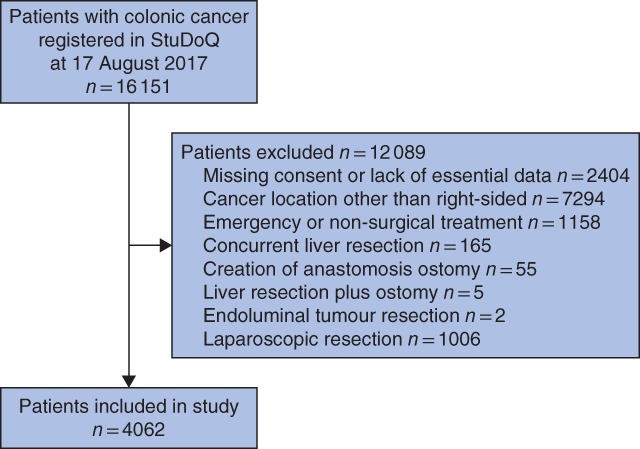
Patient selection

Of 16 151 patients registered in StuDoQ|ColonCancer from January 2011 to August 2017, 4062 underwent elective open right hemicolectomy (*Fig*. 1); 2742 (67·5 per cent) had a handsewn and 1320 (32·5 per cent) a stapled anastomosis. Preoperative characteristics of the two groups were similar, with the exception of older age in the stapled group (mean 73·9 years *versus* 72·9 years in the handsewn group; *P* = 0·020), more patients with metastatic disease (7·9 *versus* 5·3 per cent respectively; *P* = 0·001) and less likelihood of being functionally independent (Eastern Cooperative Oncology Group grade 0–1: 86·5 *versus* 88·0 per cent; *P* = 0·024) (*Table* 1).

**Table 1:** Preoperative patient characteristics according to anastomosis technique

	Handsewn (*n* = 2742)	Stapled (*n* = 1320)	*P* †
Age (years)*	72·9(10·9)	73·9(10·6)	0·020‡
Sex ratio (M : F)	1293 : 1449	622 : 698	0·980
BMI (kg/m^2^)*	26·8(5·1)	26·7(5·4)	0·380‡
Smoker	197 of 2496 (7·9)	78 of 1151 (6·8)	0·230
ASA grade			0·440
I	125 (4·6)	57 (4·3)	
II	1173 (42·8)	548 (41·5)	
III	1335 (48·7)	669 (50·7)	
IV	107 (3·9)	43 (3·3)	
V	2 (0·1)	3 (0·2)	
ECOG functional status			0·024
0–1 (independent)	2412 (88·0)	1142 (86·5)	
2–3 (partially dependent)	293 (10·7)	144 (10·9)	
4 (totally dependent)	37 (1·3)	34 (2·6)	
Co‐morbidity			
Diabetes (types 1 and 2)	638 (23·3)	330 (25·0)	0·320
Hypertension	1831 (66·8)	904 (68·5)	0·280
History of severe COPD	182 (6·6)	98 (7·4)	0·360
Chronic steroid use	37 (1·3)	25 (1·9)	0·190
Dialysis	24 (0·9)	12 (0·9)	0·730
Disseminated metastatic cancer	144 (5·3)	104 (7·9)	0·001
Weight loss (> 10% bodyweight)	329 (12·0)	177 (13·4)	0·190
Alcohol abuse (ICD F10·1)	84 (3·1)	39 (3·0)	0·850
UICC stage	(*n* = 2730)	(*n* = 1307)	
1	571 (20·9)	304 (23·3)	0·110
2	1056 (38·7)	490 (37·5)	
3	788 (28·9)	344 (26·3)	
4	315 (11·5)	169 (12·9)	
pT category	(*n* = 2735)	(*n* = 1317)	0·540
T0–2	660 (24·1)	340 (25·8)	
T3–4	2075 (75·9)	977 (74·2)	
pN category	(*n* = 2735)	(*n* = 1317)	0·220
N0	1675 (61·2)	833 (63·2)	
N1–2	1060 (38·8)	484 (36·8)	

Values in parentheses are percentages unless indicated otherwise;

* values are mean(s.d.). ECOG, Eastern Cooperative Oncology Group; COPD, chronic obstructive pulmonary disease.

† χ^2^ test, except

‡ Mann–Whitney *U* test.

Patients receiving a stapled anastomosis were more likely to undergo a midline laparotomy than those having a handsewn anastomosis (62·4 *versus* 69·0 per cent respectively; *P* < 0·001) and less likely to have CME (84·0 *versus* 80·8 per cent; *P* = 0·016) (*Table* 2). Duration of surgery was significantly shorter for the stapled anastomosis group (mean(s.d.) 120·5(46·5) *versus* 134·1(49·0) respectively; *P* < 0·001). LOS for the handsewn and stapled groups (13·4(9·2) *versus* 13·6(9·5) days respectively; *P* = 0·700) and procedure‐related hospital readmission rates (4·9 *versus* 4·4 per cent; *P* = 0·490) did not differ between the groups.

**Table 2:** Surgical characteristics

	Handsewn (*n* = 2742)	Stapled (*n* = 1320)	*P* †
Duration of surgery (min)*	134·1(49·0)	120·5(46·5)	< 0·001‡
Extended resection	380 (13·9)	176 (13·3)	0·650
Laparotomy	(*n* = 2473)	(*n* = 1227)	< 0·001
Median	1543 (62·4)	846 (69·0)	
Transverse	930 (37·6)	381 (31·1)	
Complete mesocolic excision	(*n* = 2413)	(*n* = 1218)	0·016
Yes	2027 (84·0)	984 (80·8)	
No	386 (16·0)	234 (19·2)	
Duration of hospital stay (days)*	13·4(9·2)	13·6(9·5)	0·700‡
30‐day mortality	80 (2·9)	48 (3·6)	0·220
MTL30‐positive^16^	253 (9·2)	132 (10·0)	0·430

Values in parentheses are percentages unless indicated otherwise;

* values are mean(s.d.).

† χ^2^ test, except

‡ Mann–Whitney *U* test.

The 30‐day rate of postoperative incisional SSI, anastomotic leakage and death for all patients was 9·9, 3·6 and 3·2 per cent respectively. No significant difference was found in the 30‐day postoperative mortality rate between the two groups: 2·9 per cent for handsewn *versus* 3·6 per cent for stapled anastomosis (*P* = 0·220). The groups did not differ with regard to rates of surgical complications such as SSI (10·2 *versus* 9·5 per cent respectively; *P* = 0·450), anastomotic leakage (3·9 *versus* 3·0 per cent; *P* = 0·130), postoperative ileus (4·0 *versus* 3·6 per cent; *P* = 0·520) or other surgical complications (7·6 *versus* 7·2 per cent; *P* = 0·660) (*Table* 3).

**Table 3:** Unadjusted postoperative complications by anastomosis technique

	Handsewn (*n* = 2742)	Stapled (*n* = 1320)	*P* †
Anastomotic leak	106 (3·9)	40 (3·0)	0·130
Postoperative ileus	111 (4·0)	48 (3·6)	0·520
Return to operating room	264 (9·6)	139 (10·5)	0·640
Superficial site infection	280 (10·2)	125 (9·5)	0·450
Postoperative bleeding	49 (1·8)	21 (1·6)	0·650
Clavien–Dindo grade			1·000
0–IIIa	2383 (86·9)	1147 (86·9)	
IIIb–V	359 (13·1)	173 (13·1)	
Clavien–Dindo grade			0·002
0	1699 (62·0)	880 (66·7)	
I–II	550 (20·1)	207 (15·7)	
III–V	493 (18·0)	233 (17·7)	

Values in parentheses are percentages.

† χ^2^ test.

Univariable analysis of postoperative complications according to the Clavien–Dindo classification revealed small differences between the two groups, with more minor complications (grade I–II) in the handsewn than in the stapled group (20·1 *versus* 15·7 per cent respectively; *P* < 0·002). No significant differences were observed in major surgical complications: reoperation rate (9·6 *versus* 10·5 per cent; *P* = 0·640) or postoperative bleeding (1·8 *versus* 1·6 per cent; *P* = 0·650). In addition, neither the 30‐day mortality rate (2·9 *versus* 3·6 per cent; *P* = 0·220) nor MTL30‐positive status (9·2 *versus* 10·0 per cent; *P* = 0·430) differed between the groups.

**Figure 2:**
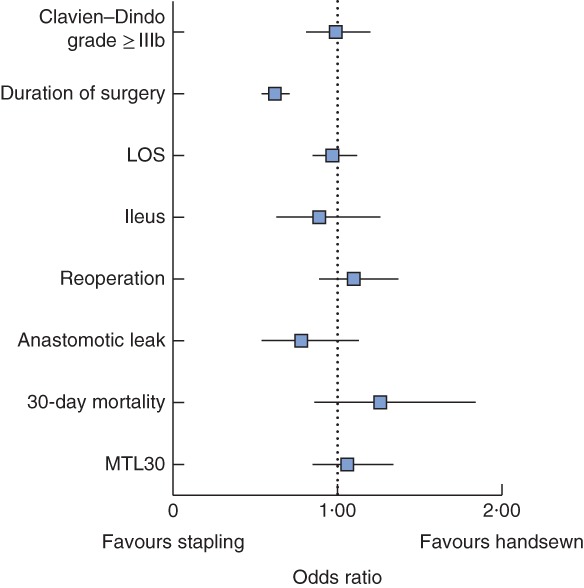
Forest plot of various outcomes by type of anastomosis. Odds ratios are shown with 95 per cent confidence intervals. LOS, length of postoperative hospital stay

In multivariable analysis, the odds ratio (OR) for stapled anastomosis was associated with a significant reduction in duration of surgery (OR 0·62, 95 per cent c.i. 0·54 to 0·71) but had no impact on the primary endpoint anastomotic leakage (OR 0·78, 0·54 to 1·13) or the secondary endpoints: reoperation rate (OR 1·10, 0·89 to 1·37), postoperative ileus (OR 0·89, 0·63 to 1·26), Clavien–Dindo grade IIIb or above (OR 0·99, 0·81 to 1·20) or LOS (OR 0·97, 0·85 to 1·12), 30‐day mortality (OR 1·26, 0·86 to 1·84) or MTL30‐positive status (OR 1·06, 0·85 to 1·34) (*Fig*. 2; *Tables* 4, 5, 6).

**Table 4:** Multivariable best‐fit model for the outcomes Clavien–Dindo grade, duration of surgery and length of hospital stay

	Clavien–Dindo grade ≥ IIIb		Duration of surgery		Length of hospital stay	
	Odds ratio	*P*	Odds ratio	*P*	Odds ratio	*P*
Type of anastomosis						
Handsewn	1·00 (reference)		1·00 (reference)		1·00 (reference)	
Stapled	0·99 (0·81, 1·2)	0·880	0·62 (0·54, 0·71)	< 0·001	0·97 (0·85, 1·12)	0·710
Type of procedure						
Hemicolectomy	1·00 (reference)		1·00 (reference)		1·00 (reference)	
Extended hemicolectomy	1·03 (0·78, 1·35)	0·850	1·44 (1·20, 1·73)	< 0·001	1·07 (0·89, 1·3)	0·470
ASA I (per each additional ASA category)	1·85 (1·58, 2·18)	< 0·001	1·09 (0·98, 1·22)	0·110	1·62 (1·45, 1·81)	< 0·001
BMI (per 5 kg/m^2^)	1·12 (1·03, 1·22)	0·008	1·25 (1·18, 1·33)	< 0·001	1·08 (1·01, 1·5)	0·019
Age (per 10 years)	1·13 (1·02, 1·25)	0·020	0·94 (0·89,1·00)	0·042	1·34 (1·25, 1·43)	< 0·001

Values in parentheses are 95 per cent confidence intervals.

**Table 5:** Multivariable best‐fit model for the outcomes anastomotic leak, postoperative ileus and reoperation

	Anastomotic leak		Postoperative ileus		Reoperation	
	Odds ratio	*P*	Odds ratio	*P*	Odds ratio	*P*
Type of anastomosis						
Handsewn	1·00 (reference)		1·00 (reference)		1·00 (reference)	
Stapled	0·78 (0·54, 1·13)	0·200	0·89 (0·63, 1·26)	0·520	1·10 (0·89, 1·37)	0·380
Type of procedure						
Hemicolectomy	1·00 (reference)		1·00 (reference)		1·00 (reference)	
Extended hemicolectomy	1·18 (0·75, 1·86)	0·480	1·08 (0·69, 1·70)	0·720	1·14 (0·85, 1·53)	0·370
ASA I (per each additional ASA category)	1·87 (1·42, 2·48)	< 0·001	1·34 (1·04, 1·71)	0·220	1·59 (1·35, 1·87)	< 0·001
BMI (per 5 kg/m^2^)	1·03 (0·89, 1·21)	0·680	1·12 (0·97, 1·29)	0·110	1·18 (1·08, 1·29)	< 0·001
Age (per 10 years)	0·85 (0·72, 1·00)	0·049	0·99 (0·84, 1·17)	0·940	1·00 (0·89, 1·12)	0·980

Values in parentheses are 95 per cent confidence intervals.

**Table 6:** Multivariable best‐fit model for the outcomes 30‐day mortality and MTL30‐positive status

	30‐day mortality		MTL30‐positive^16^	
	Odds ratio	*P*	Odds ratio	*P*
Type of anastomosis				
Handsewn	1·00 (reference)		1·00 (reference)	
Stapled	1·26 (0·86, 1·84)	0·230	1·06 (0·85, 1·34)	0·590
Type of procedure				
Hemicolectomy	1·00 (reference)		1·00 (reference)	
Extended hemicolectomy	1·41 (0·86, 2·31)	0·170	0·92 (0·66, 1·27)	0·590
ASA I (per each additional ASA category)	3·58 (2·59, 4·95)	< 0·001	2·45 (2·02, 2·96)	< 0·001
BMI (per 5 kg/m^2^)	0·97 (0·81, 1·16)	0·730	1·08 (0·97, 1·19)	0·150
Age (per 10 years)	1·65 (1·30, 2·09)	< 0·001	1·30 (1·14, 1·47)	< 0·001

Values in parentheses are 95 per cent confidence intervals.

## Discussion

The optimal anastomosis technique after right hemicolectomy is still a matter for debate^8^. Most trials^17^, ^18^, ^19^, ^20^ are too small to provide definitive conclusions regarding the various techniques, or focus on Crohn's disease where the patient's postoperative course may be influenced by the underlying disease.

A large multicentre study^21^ analysing 999 patients who underwent ileocolic anastomosis between 2002 and 2007, mainly for colonic cancer (95·8 per cent) found that patients with a handsewn anastomosis had a significantly higher leak rate (4·9 per cent) than patients with a stapled anastomosis (2·5 per cent). A meta‐analysis^8^ also found a lower anastomotic leak rate for stapled anastomoses, but no differences in other outcomes. Conversely, stapled anastomosis was identified as an independent risk factor for anastomotic leakage in a study from Denmark^5^. In a recent multinational snapshot audit^22^ of anastomosis following right‐sided colonic resection for colonic cancer or inflammatory bowel disease, the use of staplers was identified as an independent risk factor for anastomotic leakage.

The present study offers the first registry‐based analysis of the impact of anastomosis technique (stapled *versus* handsewn) following oncological right hemicolectomy. The two techniques appear equally safe in terms of anastomotic leakage, reoperation rate, postoperative ileus, SSI and other surgical complications.

The primary endpoint of anastomotic leak rate in this study of 3·6 per cent was below the 6·4–7·5 per cent reported by Bakker and colleagues^5^ and Krarup and co‐workers^4^. This lower rate may be attributable to the fact that patients were registered at specialized cancer centres, with some bias for this population. The German Cancer Society certificates specialized cancer centres on the basis of several parameters, of which anastomotic leakage is one. Hospitals can use their own submitted data set to StuDoQ for quality control and certification, thereby perhaps creating a documentation bias.

The 13‐day LOS reported in this study is considerably longer than that of studies on colectomy from the American College of Surgeons National Surgical Quality Improvement Program (ACS NSQIP) registry^23^. This may be due to less economic pressure to reduce LOS, slower implementation of enhanced recovery regimens in clinical practice, and the higher rate of CME. The rate of postoperative ileus in the present study was surprisingly low compared with that of the ACS NSQIP registry^23^ ^24^ (3·9 *versus* 12·7 per cent respectively). This difference could also be a documentation bias, as postoperative ileus has not been defined precisely. Additionally, the rate of CME was higher in the handsewn group, which may account for the higher rate of minor (grade I–II) complications, as it has been shown^10^ that CME resection is associated with more, and more severe, complications than non‐CME resection.

Limitations of the present study include the lack of cost analysis and information missing in the StuDoQ registry, such as end‐to‐end or side‐to‐side connections and details of handsewn techniques and materials used. Although there was no difference in the short term, it is unclear whether the two methods differ in the long term with regard to stenosis rate, oncological outcome or quality of life.

Stapled and handsewn techniques for creating ileocolic anastomoses after open oncological right hemicolectomy seem equally safe and effective in the short term. Stapling was associated with reduced duration of surgery and fewer minor (grade I–II) complications.

## Supporting information


**Table S1** All participating hospitals and surgical directors who contributed patient data to the StuDoQ|ColonCancer registry

## References

[1] Siegel RL , Miller KD , Fedewa SA , Ahnen DJ , Meester RGS , Barzi A *et al* Colorectal cancer statistics, 2017. CA Cancer J Clin 2017; 67: 177–193. 28248415 10.3322/caac.21395

[2] Brenner H , Kloor M , Pox CP . Colorectal cancer. Lancet 2014; 383: 1490–1502. 24225001 10.1016/S0140-6736(13)61649-9

[3] Rogers SO Jr , Wolf RE , Zaslavsky AM , Wright WE , Ayanian JZ . Relation of surgeon and hospital volume to processes and outcomes of colorectal cancer surgery. Ann Surg 2006; 244: 1003–1011. 17122626 10.1097/01.sla.0000231759.10432.a7 PMC1856632

[4] Krarup PM , Jorgensen LN , Andreasen AH , Harling H ; Danish Colorectal Cancer Group . A nationwide study on anastomotic leakage after colonic cancer surgery. Colorectal Dis 2012; 14: e661–e667. 22564292 10.1111/j.1463-1318.2012.03079.x

[5] Bakker IS , Grossmann I , Henneman D , Havenga K , Wiggers T . Risk factors for anastomotic leakage and leak‐related mortality after colonic cancer surgery in a nationwide audit. Br J Surg 2014; 101: 424–432. 24536013 10.1002/bjs.9395

[6] Hansen O , Schwenk W , Hucke HP , Stock W . Colorectal stapled anastomoses. Experiences and results. Dis Colon Rectum 1996; 39: 30–36. 8601353 10.1007/BF02048265

[7] Kano M , Hanari N , Gunji H , Hayano K , Hayashi H , Matsubara H . Is ‘functional end‐to‐end anastomosis’ really functional? A review of the literature on stapled anastomosis using linear staplers. Surg Today 2017; 47: 1–7. 26988855 10.1007/s00595-016-1321-9

[8] Choy PY , Bissett IP , Docherty JG , Parry BR , Merrie A , Fitzgerald A . Stapled *versus* handsewn methods for ileocolic anastomoses. Cochrane Database Syst Rev 2011; (9)CD004320. 10.1002/14651858.CD004320.pub3 21901690

[9] Gustafsson P , Jestin P , Gunnarsson U , Lindforss U . Higher frequency of anastomotic leakage with stapled compared to hand‐sewn ileocolic anastomosis in a large population‐based study. World J Surg 2015; 39: 1834–1839. 25708508 10.1007/s00268-015-2996-6

[10] Bertelsen CA , Neuenschwander AU , Jansen JE , Kirkegaard‐Klitbo A , Tenma JR , Wilhelmsen M *et al*; Copenhagen Complete Mesocolic Excision Study (COMES); Danish Colorectal Cancer Group (DCCG) . Short‐term outcomes after complete mesocolic excision compared with ‘conventional’ colonic cancer surgery. Br J Surg 2016; 103: 581–589. 26780563 10.1002/bjs.10083

[11] Hohenberger W , Weber K , Matzel K , Papadopoulos T , Merkel S . Standardized surgery for colonic cancer: complete mesocolic excision and central ligation – technical notes and outcome. Colorectal Dis 2009; 11: 354–364. 19016817 10.1111/j.1463-1318.2008.01735.x

[12] van Rooijen SJ , Jongen AC , Wu ZQ , Ji JF , Slooter GD , Roumen RM *et al* Definition of colorectal anastomotic leakage: a consensus survey among Dutch and Chinese colorectal surgeons. World J Gastroenterol 2017; 23: 6172–6180. 28970733 10.3748/wjg.v23.i33.6172 PMC5597509

[13] Bruce J , Krukowski ZH , Al‐Khairy G , Russell EM , Park KG . Systematic review of the definition and measurement of anastomotic leak after gastrointestinal surgery. Br J Surg 2001; 88: 1157–1168. 11531861 10.1046/j.0007-1323.2001.01829.x

[14] Kirchhoff P , Clavien PA , Hahnloser D . Complications in colorectal surgery: risk factors and preventive strategies. Patient Saf Surg 2010; 4: 5. 20338045 10.1186/1754-9493-4-5 PMC2852382

[15] Dindo D , Desmartines N , Clavien PA . Classification of surgical complications: a new proposal with evaluation in a cohort of 6336 patients and results of a survey. Ann Surg 2004; 240: 205–213. 15273542 10.1097/01.sla.0000133083.54934.ae PMC1360123

[16] Wiegering A , Wellner U , Seyfried F , Hardt J , Klinger C , Buhr H *et al* [MTL30 as surrogate parameter for quality of surgically treated diseases: establishment based on the StuDoQ register of the German Society for General and Visceral Surgery.] Chirurg 2017; 88: 977–982. 28761965 10.1007/s00104-017-0479-z

[17] Kracht M , Hay JM , Fagniez PL , Fingerhut A . Ileocolonic anastomosis after right hemicolectomy for carcinoma: stapled or hand‐sewn? A prospective, multicenter, randomized trial. Int J Colorectal Dis 1993; 8: 29–33. 8492040 10.1007/BF00341273

[18] Anwar S , Hughes S , Eadie AJ , Scott NA . Anastomotic technique and survival after right hemicolectomy for colorectal cancer. Surgeon 2004; 2: 277–280. 15570847 10.1016/s1479-666x(04)80097-0

[19] Yamamoto T , Bain IM , Mylonakis E , Allan RN , Keighley MR . Stapled functional end‐to‐end anastomosis *versus* sutured end‐to‐end anastomosis after ileocolonic resection in Crohn disease. Scand J Gastroenterol 1999; 34: 708–713. 10466883 10.1080/003655299750025921

[20] Muñoz‐Juárez M , Yamamoto T , Wolff BG , Keighley MR . Wide‐lumen stapled anastomosis *vs*. conventional end‐to‐end anastomosis in the treatment of Crohn's disease. Dis Colon Rectum 2001; 44: 20–25. 11805559 10.1007/BF02234814

[21] Puleo S , Sofia M , Trovato MA , Pesce A , Portale TR , Russello D *et al* Ileocolonic anastomosis: preferred techniques in 999 patients. A multicentric study. Surg Today 2013; 43: 1145–1149. 23111464 10.1007/s00595-012-0381-8

[22] 2015 European Society of Coloproctology collaborating group . Relationship between method of anastomosis and anastomotic failure after right hemicolectomy and ileo‐caecal resection: an international snapshot audit. Colorectal Dis 2017; 19: e296–e311. 10.1111/codi.13646 28263043

[23] Althumairi AA , Canner JK , Pawlik TM , Schneider E , Nagarajan N , Safar B *et al* Benefits of bowel preparation beyond surgical site infection: a retrospective study. Ann Surg 2016; 264: 1051–1057. 26727098 10.1097/SLA.0000000000001576

[24] Moghadamyeghaneh Z , Hwang GS , Hanna MH , Phelan M , Carmichael JC , Mills S *et al* Risk factors for prolonged ileus following colon surgery. Surg Endosc 2016; 30: 603–609. 26017914 10.1007/s00464-015-4247-1

